# The value of the WIRHE Scholarship Programme in training health professionals for rural areas: Views of participants

**DOI:** 10.4102/phcfm.v9i1.1488

**Published:** 2017-10-13

**Authors:** Nontsikelelo O. Mapukata, Ian Couper, Jocelyn Smith

**Affiliations:** 1 Centre for Rural Health, Faculty of Health Sciences, University of the Witwatersrand, South Africa; 2 Ukwanda Centre for Rural Health, Faculty of Medicine and Health Sciences, Stellenbosch University, South Africa

## Abstract

**Introduction:**

Rural hospitals in South Africa, as elsewhere, face enduring shortages of, and challenges in attracting and retaining, suitably qualified staff. The Wits Initiative for Rural Health Education (WIRHE), based at the University of the Witwatersrand but covering three universities, is a rural scholarship programme established to find local solutions to these challenges in the North West and Mpumalanga provinces. The purpose of this evaluation was to ascertain whether the WIRHE project was achieving its objectives.

**Methods:**

This article draws from an evaluation commissioned by the Swiss-South African Cooperative Initiative, a major funder of the programme when WIRHE was launched in 2003. Qualitative interviews were conducted either as face-to-face meetings or telephonically with 21 WIRHE students and graduates. Content analysis was undertaken to identify common themes.

**Results:**

There was a consistency in the findings as the students and graduates reported similar experiences. Many of the participants were overwhelmed by their initial challenges of having to adapt to a different language, an institutional culture and resources that they previously did not have access to. The participants acknowledged the role of WIRHE staff in facilitating the transition from home to university and, in particular, the value of the financial and academic support. The geographic distance to Wits presented a challenge for the Pretoria- and Sefako Makgatho-based students. The holiday work affirmed clinical advantages for WIRHE students and heightened students’ interest in becoming healthcare workers.

**Conclusion:**

WIRHE’s key success factors are the financial, academic and emotional support offered to students. WIRHE achieved its objectives based on a principled strategic approach and an understanding that students from rural backgrounds are more likely to return to rural areas. The study supports the value of structured support programmes for students of rural origin as they pursue their studies.

## Introduction

Students of rural origin often have to deal with a range of challenges in accessing institutions of higher learning.^1,2^ Even when they are in good academic standing, the social status of their families is often a hindrance in them pursuing professional degrees of their choice.^1^ A growing body of knowledge from around the world attests to the fact that students of rural origin have a greater propensity to return to work in their region of origin than do students from an urban background.^3,4,5^ Similar findings have been demonstrated by researchers in South Africa.^6,7^

The Wits Initiative for Rural Health Education (WIRHE), a project of the Centre for Rural Health at the University of the Witwatersrand (Wits), is a rural scholarship programme established to find local solutions to some of the human resource challenges in the North West and Mpumalanga provinces. WIRHE started in 2003, based on the assumptions that, with the right support, disadvantaged students from underserved, rural areas in the North West and Mpumalanga provinces would be able to enter health professions training at university, succeed with their studies, and then work back their scholarship commitments in health facilities in the places from which they are drawn. The expectation is that a proportion would continue to serve in these areas after completing that commitment. WIRHE drew from the initial experiences of the Friends of Mosvold scholarship scheme (now called Umthombo Youth Development Foundation [UYDF]), a similar initiative in KwaZulu-Natal,^8^ in developing its model.

Both scholarship programmes are premised on the belief that, despite the enormous historical and educational disadvantages rural people face in accessing and succeeding at tertiary education, there are young people with potential that have ties to such areas and an interest in working there. With the right kind of financial and other assistance, such youth could qualify as health professionals, work in their own local areas, and begin to fill the gaps in rural hospitals. Although funding scholarships for rural school leavers is only one aspect of what is needed, it is seen as an effective entry point and is recommended as an intervention by the World Health Organization in its guidelines for increasing access to health workers in rural areas.^9^

This article draws from an evaluation commissioned by the Swiss-South Africa Cooperation Initiative (SSACI), a major funder of the WIRHE programme. The purpose of the evaluation was to ascertain whether WIRHE achieved its objectives, how well it did this, whether there was a continuing need for such an initiative, and what could be learned from the experience. The focus of this article is to report on the perceived value of the WIRHE scholarship as reported by participants, namely students and graduates.

## Methods

Ten years after the launch of the WIRHE programme, students and graduates were tracked down from the project’s database. All 27 students and graduates who had received funding from SSACI at that time were targeted, but the timing of the evaluation (when many students had already left for vacation) made it difficult to contact all, and thus 6 of the group could not be interviewed. Where possible semi-structured face-to-face interviews were conducted, but logistical issues (where graduates were working and where students were based for their vacations) meant that 12 of these were conducted telephonically. The interviews were conducted by an external evaluator (J.S.). The focus of this article is on the responses to three of the probing questions that were presented to the students and the graduates, namely:

Describe your experiences when you started university. What were the highlights? What were some of the challenges you faced?

What support does or did the scholarship programme offer you?

Can you describe some of your holiday experiences working in hospitals? What kind of relationship do you have with that hospital?

### Ethical considerations

The interviews were audio-recorded, transcribed and reviewed. Content analysis was then carried out, based on an interpretative paradigm, using the filter of the purpose of the evaluation, and common themes were identified. The anonymised interviews and the themes were reviewed by the co-authors (I.C., N.M.) and consensus was reached. Informed consent was obtained from the participants prior to the interviews, and anonymity was preserved with only the external evaluator (J.S.) being aware of which participants were interviewed. Permission was granted for the study by the Human Research Ethics Committee of the University of the Witwatersrand (M10303). It is part of a broader research programme to evaluate the WIRHE scholarship.

## Results

All 21 students and graduates contacted, representing the majority of the 27 WIRHE students funded by SSACI, agreed to be interviewed. There were 12 women and 9 men, of whom 6 were graduates. The remaining 15 at that time were still students, in their senior years. Nine were from Mpumalanga and the remainder from the North West Province. They were studying, or had completed, the following courses: medicine (10), occupational therapy (5), pharmacy (3), physiotherapy (2) and nursing (1). They were attending or had attended the following institutions: Wits (15), SefakoMakgatho University (SMU), previously known as University of Limpopo Medunsa Campus (5), and University of Pretoria (UP) (1). All the verbatim quotes included in this section reflect the professional categories of the graduates (MD = Doctor: P = Pharmacist; PT = Physiotherapist; OT = Occupational Therapist; N = Nurse) as well as their corresponding provincial allocation (MP = Mpumalanga or NW = North West). Quotes from students are represented with letter S and the name of the corresponding university. Thus, S-W is a student who was based at Wits, whilst S-SMU is a student who was studying at SMU.

The emerging themes from the analysis are presented below.

### Coping with university

Challenges experienced by rural students attending university for the first time were many. Students admitted to feeling overwhelmed:

‘I missed orientation week. My mother had not yet found the money for registration. I didn’t know where everything was. Coming from a small place, Johannesburg was quite scary.’ (PT-NW)

Issues of adjustment were described as those that other class - mates would not understand – which made the WIRHE student feel very alone: ‘Joburg felt like a jungle, and Wits made it worse’ (MD-MP).

There was a prevailing feeling that SMU was a less stressful environment compared to Wits: ‘there are not so many distractions, and it is not as competitive’ (S-SMU).

English proved to be a challenge for many of the WIRHE students. There were those who were concerned about the lecturers’ accents that were difficult to understand and the challenge of having to do everything in English: ‘everything took much longer because I had to use a dictionary all the time’ (S-W).

Another explained: ‘the school I came from taught in vernacular, so I was scared to ask questions in the first few months as I was not sure if I made sense’ (OT-MP).

This was not just about language ability; a medical student who was a national debater whilst at school did not participate in class for fear of being judged: ‘For 4 years I did not ask questions in class – I was afraid people would laugh at me. I was able to talk more in smaller groups’ (S-W).

A different language challenge was described at Medunsa, by a Setswana-speaking student who found that there were not many who spoke his language: ‘there were not that many Tswana speakers. They spoke a variety of languages – Shangaan, Sepedi and Zulu. In first year, I communicated with my friends in English’ (S-SMU).

The graduates felt that they were not prepared, at school level, for university, particularly the level of English that was required. A number of participants had never used a computer before coming to university. Learning in English, using computers, and adjusting to the academic requirements of university were difficult: ‘it was a shock when I had to write a 1000 word essay in English, and type it’ (OT-MP).

There was also the challenge of adjusting from a rural area to Johannesburg which was perceived to be a fast-paced environment: ‘I was not used to how fast Johannesburg was. … I had to learn to use the libraries and computers’ (P-NW).

One student acknowledged the opportunity this presented: ‘Lectures were in English and mine improved drastically during this time’ (PT-MP).

### Supportive role of Wits Initiative for Rural Health Education

Common amongst the students was the anxiety that was associated with everything being new: ‘adjusting to the new surrounding and work was stressful. I had to read much more than I had to in school’ (S-SMU).

The role of WIRHE was informed by the participants’ experiences at the point of entry, during the adjustment period as they socialised to their programmes and the community: ‘University was crazy. I have never seen tall buildings like that before, and so many people’ (MD-MP).

The students had left their home and families for the first time, to take up residency in unfamiliar dwellings: ‘city life seemed frightening and overwhelming’ (PT-MP).

They were amongst strangers: ‘it was the first time I lived away from home’ (OT-MP).

A medical student related that the only person she knew when she arrived at Wits was the driver from the Department of Health who had brought her in: ‘WIRHE staff showed me where to go and helped me find accommodation’ (S-W).

In many ways, the programme fulfilled a parental role for many of the students: ‘the scholarship offered a proper support, not only money’ (P-NW).

Students felt that WIRHE staff members were the people to go to whenever they felt overwhelmed and needed advice. ‘It has been almost like a shoulder to cry on’ (S-W).

Students commented that WIRHE mediated many of the issues on their behalf and facilitated a sense of belonging: ‘I did not know anyone, and WIRHE performed the role of a family for me’ (PT-MP).

### Academic support

WIRHE was seen to provide emotional support that assisted with adjustment and thus enabled students to cope: ‘WIRHE programme helped emotionally and financially’ (S-W).

The programme organised counselling for students in need, and academic support: ‘they arranged someone to help me when my mother passed away’ (OT-MP).

Mentoring was fairly individual, with WIRHE staff intervening as needed: ‘the programme helped me stay at university and stick it out’ (S-W).

One of the graduates explained that two of his friends who were on provincial bursaries dropped out because of the fact that they had no support. ‘Had they been supported by WIRHE, I am convinced they would have been able to complete their medical degrees’ (MD –MP).

Other graduates confirmed this thinking as they felt that they would not have completed their degrees without the support of the programme, particularly the mentors. ‘The support was wonderful’ (MD-NW).

WIRHE staff arranged compulsory meetings with students from all the three universities and tried to get them to identify issues early, so that timely support could be provided. Students acknowledged the value of the group meetings held twice a year, where students from all courses and campuses met together and shared issues of common interest. Students found the networking valuable, saying, ‘meeting with other scholarship students helped’ (S-W), especially when they met students studying the same courses at different universities. It helped them realise that their experiences were not unique:

‘When you hear other students talking of their challenges, you find that they are exactly the same as yours. You feel like you can do this, there is always someone before you who has done it. It really helps.’ (S-SMU)

Being university-based, WIRHE was able to organise tutoring from the relevant departments when students needed specific academic support, at least at Wits, where students dropped in regularly at the WIRHE office. Some students at the other two campuses felt that although WIRHE staff members always reached out to them telephonically, ‘it was easier for the Wits based students to access this resource’ (S-UP).

### Financial and logistic support

The scholarship programme relieved students of worrying about paying fees and accommodation.

‘I heard about the WIRHE programme at Tintswalo hospital. I was already at Wits doing my orientation week when I heard from WIRHE that I have been accepted. It was a huge relief.’ (S-W)

For many WIRHE was seen to arrange everything for them. One said that ‘the scholarship “saved me” because “it helped with fees, accommodation and everything I needed”’ (P-NW).

One graduate described his joy when, 2 days before registration, he received a letter from WIRHE confirming his receipt of the scholarship: ‘until then, I had been waiting for a miracle. I knew I only had R2000 for registration. A number of students reported similar experiences’ (PT-MP).

Students reported that WIRHE arranged for them to buy textbooks and stationery with a private supplier. Where students found that fees were not paid, they called the WIRHE offices and received help: ‘the WIRHE scholarship was God-sent; they gave me everything, even an allowance’ (PT-NW).

The students were grateful that they did not have to worry about finances:

‘When I received the scholarship, the stress went away and I could focus on our studies. I could now get the textbooks I needed. Before that I had been using outdated books or had to borrow and photocopy textbooks.’ (S-SMU)

A student said that knowing fees were being paid in full brought much relief: ‘without the financial support and mentoring, I do not believe that I would have gotten my degree’ (N-NW).

Not having to stress about the financial burden encouraged one student to do his best: ‘make a difference in someone else’s life’ (PT-MP).

### The value of holiday work

Holiday work, or community service in the students’ home districts, was facilitated through the WIRHE office. Many of the students acknowledged that before being admitted to the scholarship there was no motivation for them to visit a health facility: ‘The first 2 years, I did not do holiday work, as I was not on the bursary’ (MD-NW).

Holiday work exposed students to genuine experiences, and practical examples of what they would encounter during their university years:

‘I learned to clerk patients, take histories, and was expected to observe delivery of babies. When I came back to university in 4th year, I had learned all those things, so the block was easy for me, and I did very well.’ (MD-NW)

Most students referred to their holiday work as a highlight of the WIRHE programme, ‘I learned a lot. … Staff was helpful. They encouraged us to ask questions’ (S-SMU).

They often developed a relationship with the hospital and with other students there, and gained confidence in working in a rural setting: ‘I have been working in one hospital for a couple of years now. The staff are all familiar to me and the experience has been good’ (S-UP).

For one student it affirmed his career choice: ‘It confirmed that I was doing the right thing, even though physiotherapy had not been my first choice’ (PT-MP).

One student enjoyed it so much that he said: ‘I spent much longer there than I was supposed to’ (MD-NW).

Another said that serving the community in this way changed his perspective, because ‘I saw that when I went back I gained respect and this made me want to give back to my community’ (OT-MP).

One of the challenges regarding holiday work was that supervision of students was variable. Students sometimes arrived and no one knew what they were doing there. Some students found staff too busy to help, but then some hospitals were more willing to take on students than others: ‘it all depended where you went’ (S-W).

One graduate reflected on his student experience as a period of no growth even though he enjoyed interacting with patients performing unrelated activities: ‘Working at the same clinic, every year for my holiday experience. … I did not always do OT-related things, but I enjoyed it. Sometimes I took patients’ vital signs …’ (OT-NW).

A few, mostly medical students or graduates, mentioned that in their first or second year they did not find it that useful, as doctors and nurses often found them an irritation. A medical student explained that, though the holiday work did not help her studies directly as she was based in a clinic, ‘the experience allowed me to feel like I was fulfilling my passion’ (S-W).

However, once their clinical skills improved they found it more beneficial: ‘by the time I returned in January for my 4th year, I had advantage over the other students. It prepared me well for the year ahead’ (PT-NW).

This was confirmed by another student: ‘I had an advantage over my peers because I had seen things at the hospital, like a lumbar puncture. I could explain things in class …’ (MD-MP).

Students were initially apprehensive and found holiday work to be exhausting: ‘it was very tiring at first. I was not used to the working hours’ (S-SMU).

However, for most of them it turned out to be a rewarding experience: ‘the holiday work was a lot more exciting than studying!’ (S-W).

## Discussion

This study details significant findings about students’ experiences in adjusting to higher education institutions, the benefits of having financial support, onsite academic support and the value of holiday work. The results from this study provide evidence for a defined role of the WIRHE programme, as an example of a scholarship and mentoring programme, in supporting students of rural origin towards their goal of becoming healthcare professionals. Ross reported similar findings as he addressed the very same issue in a study that explored the role of Umthombo Youth Development Foundation Scholarship Scheme (UYDF SS) in supporting students of rural origin.^7^

Coping in a university was reflected on as a cross-cutting challenge as WIRHE students were either navigating spaces or negotiating a culture that were different – from the required competencies in English to a fast paced environment they could not keep up with and accents that were not familiar. Much that is written about the process students of rural origin undergo in becoming a university student validates the experiences of these WIRHE students, such as the deep-seated feeling of being out of place and not fitting in^10^ and being unprepared, academically and socially.^11^ This study has confirmed previous research that identified challenges facing students of rural origin as being multifaceted, with many of these attributed to the school years and the social profile of their families,^12^ leading to students feeling like a ‘fish out water’.^13^ The role of WIRHE in simulating a family setting is seen as moderating negative experiences associated with the adjustment process. There was consensus amongst the WIRHE students and graduates that key to their success was having access to financial support and mentoring, similar to the findings reported by Ross.^7^ Even in a well-resourced environment such as Australia, rural students are more likely to report that they feel a lot of stress than their urban counterparts, largely because of financial concerns and transitioning issues.^14^ Wilson et al. cite financial support and mentoring as critical factors in ensuring success of rural students.^3^ Whilst finance was perceived to be a timeous intervention, students had the insight to appreciate peer mentoring as a form of role modelling during the combined meetings. Mentoring is considered to be one of the key practical strategies in the recruitment and retention of students of rural origin.^6,15,16,17^ The two components (finance and mentoring) were perceived to place WIRHE students in a position of privilege compared to their counterparts, who had a similar profile but did not have access to the same resources, that is, other students of rural origin who were not funded. WIRHE students also see themselves as having a significant advantage in their clinical exposure through their holiday work that places them at a different level to most of their peers, in the application of their knowledge as they progress through their training.

Holiday work, which is a form of community service, was perceived to be a process of socialising students to the workplace that sought to simulate the apprenticeship model and to motivate affiliation to the community of origin. It was seen as a non-punitive model of upskilling WIRHE students, though a few respondents acknowledged their initial resistance, hence the reference to ‘holiday work’. Rose and Janse van Rensburg-Bonthuyzen refer to these pull factors as the intrinsic determinants that may define those who have an affinity for rural medicine.^17^ A key factor in the WIRHE programme is not only the emphasis placed on students’ responsibilities to return to their places of origin, which they clearly understand, but also the facilitation of linkages and relationships with the districts and hospitals through holiday work experiences, which are appreciated because they assist students to apply what they are learning and even give them an edge over their peers, as noted above. This form of nurturing is supported by Lubben et al. who suggest positive links between students’ career orientation and their career aspirations.^18^

The combination of these experiences ensured that students developed relationships with the public health authorities in their communities of origin, facilitating easier placement and insight into working in rural areas to fulfil their commitments after graduation.^17,19,20^ The benefits associated with such familiarity with the workplace have been described in other contexts.^21^ Although the work placements presented a challenge in the earlier years, the opportunities for students to improve skills in facilities where there was a supportive environment allowed students to define their role in the future as rural practitioners.^22^

The study confirmed that the role of the WIRHE programme was significant in the support it provided especially to the Wits based students as they had unlimited access to their mentors. This suggests that the key to the success of rural origin students is not so much related to their personal characteristics but rather to how access to learning in higher education institutions is mediated. Ultimately, the environment in medical schools needs to be transformed so that rural origin students do not have to become ‘decontextualised learners’ but are rather empowered with the knowledge and skills that will enable them to address the challenges of their contexts.^23^

### Limitations of the study

This evaluation was commissioned by a funder for their own purpose and thus only WIRHE students who were recipients of their funding were included in the sample, but there are no discernible differences between these and other WIRHE students in terms of the financial and mentoring support they receive. Secondly, not all the applicants were available for the planned face-to-face interviews because of work commitments so a number of interviews were conducted telephonically.

## Conclusion

WIRHE’s key success factors are the financial, academic and emotional support offered to students, which enable them to overcome the challenges they face in negotiating the learning environment of medical school to succeed in becoming health professional graduates ready to serve their communities. WIRHE has achieved its objectives based on a principled strategic approach that underpins the WIRHE scholarship programme and an understanding that students from rural backgrounds are more likely to return to rural areas. The study supports the value of structured support programmes for students of rural origin as they pursue their studies, together with opportunities to contextualise their learning in local communities.
